# Quartz Crystal Microbalance as a Holistic Detector for Quantifying Complex Organic Matrices during Liquid Chromatography: 1. Coupling, Characterization, and Validation

**DOI:** 10.1021/acs.analchem.3c05440

**Published:** 2024-04-29

**Authors:** Christopher Wabnitz, Aoife Canavan, Wei Chen, Mathias Reisbeck, Rani Bakkour

**Affiliations:** † TUM School of Natural Sciences, Chair of Analytical Chemistry and Water Chemistry, Technical University of Munich, Garching 85748, Germany; ‡ TUM School of Computation, Information and Technology, Heinz Nixdorf Chair of Biomedical Electronics, Technical University of Munich, Munich 81675, Germany

## Abstract

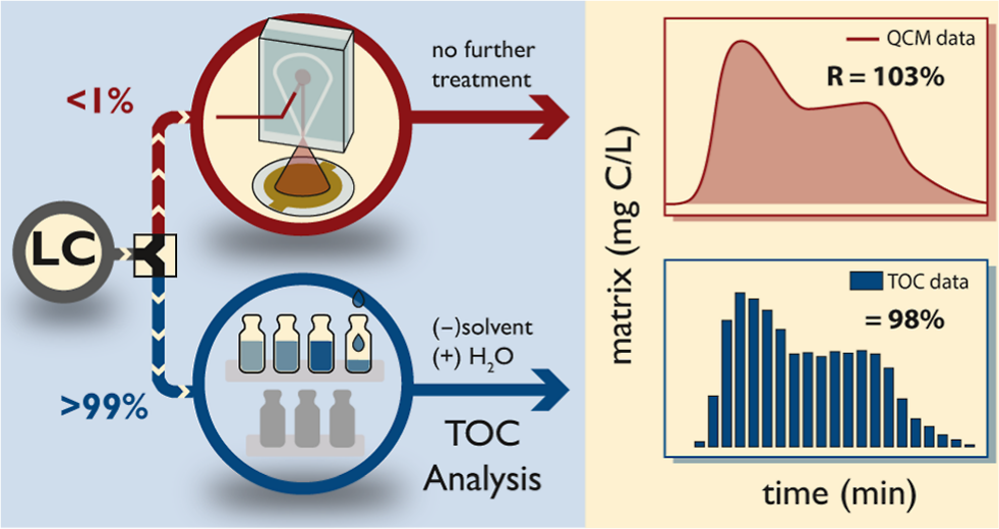

A matrix in highly complex samples can cause adverse effects on the trace analysis of targeted organic compounds. A suitable separation of the target analyte(s) and matrix before the instrumental analysis is often a vital step for which chromatographic cleanup methods remain one of the most frequently used strategies, particularly high-performance liquid chromatography (HPLC). The lack of a simple real-time detection technique that can quantify the entirety of the matrix during this step, especially with gradient solvents, renders optimization of the cleanup challenging. This paper, along with a companion one, explores the possibilities and limitations of quartz crystal microbalance (QCM) dry-mass sensing for quantifying complex organic matrices during gradient HPLC. To this end, this work coupled a QCM and a microfluidic spray dryer with a commercial HPLC system using a flow splitter and developed a calibration and data processing strategy. The system was characterized in terms of detection and quantification limits, with LOD = 4.3–15 mg/L and LOQ = 16–52 mg/L, respectively, for different eluent compositions. Validation of natural organic matter in an environmental sample against offline total organic carbon analysis confirmed the approach’s feasibility, with an absolute recovery of 103 ± 10%. Our findings suggest that QCM dry-mass sensing could serve as a valuable tool for analysts routinely employing HPLC cleanup methods, offering potential benefits across various analytical fields.

## Introduction

A challenge in trace analysis of targeted organic compounds is highly complex samples, such as environmental,^1,2^ biological,^3,4^ or food samples.^5−7^ This is the case because abundant organic and inorganic constituents of the complex sample, other than the target analyte(s) and also known as the matrix, can have a variety of adverse effects on the analytical mode of detection. These adverse effects include increased detection noise, higher detection limits, obscured peaks, false positive signals/results, signal suppression (negative matrix effect), or signal enhancement (positive matrix effect).^7,8^ Despite many instrumental developments that improved the overall detection or the separation of the sample matrix and target analytes, e.g., high-resolution mass spectrometry or multidimensional hyphenated chromatography, there are still limitations in trace analysis of targeted organic compounds due to the sample matrix.^6,7,9^ A suitable sample preparation before the instrumental analysis is often a vital key to the reduction of matrix-related adverse effects.^6,7,10^

An efficient separation of the matrix constituents from the target analyte(s) is usually the focus of such sample preparation procedures for which chromatographic cleanup methods remain one of the most often used strategies, particularly high-performance liquid chromatography (HPLC). As a stand-alone purification system or directly coupled to a detector, HPLC cleanup finds many applications mainly due to the wide variety of available column materials and modes [e.g., reversed phase (RP)],^11,12^ as well as the possibility to optimize purification using an unlimited combination of solvents.^1,11,12^ In addition to the easy automation of the sample cleanup, which increases reliability and accuracy,^13−15^ the possibility to pack columns with highly selective materials^16,17^ makes the sample preparation tunable to any target analyte of interest. Yet, the HPLC method development becomes tedious and challenging when the matrix is a complex mixture that cannot be quantified with straightforward measures. In fact, an optimal and efficient optimization of the HPLC cleanup warrants a simultaneous online quantification of both the target analyte(s) and the interfering matrix.

Numerous detectors exist for organic target analytes, yet their direct application for online quantification of organic matrices is impeded by the matrices’ heterogeneous and complex composition. Natural organic matter (NOM), for instance, comprises thousands of compounds with diverse physicochemical properties, while food matrices encompass a complex mixture of fatty acids, proteins, carbohydrates, vitamins, and more.^18,19^ Spectroscopic methods, such as fluorescence^20−24^ and UV–vis,^25−28^ have been extensively investigated for probing the structural variation of NOM. Despite their sensitivity and simplicity, these techniques are limited by the requirement for fluorophores or chromophores in all compounds, rendering them unsuitable as universal detectors for NOM.^29,30^ Additionally, their response is influenced by the chemical environment, such as pH, posing challenges for quantification.^31,32^ Similarly, mass spectrometric methods coupled with chromatography, such as gas and liquid chromatography–mass spectrometry (GC- and LC–MS) and Fourier transform ion cyclotron mass spectrometry, are highly dependent on the chemical properties of the analyte, leading to varying ionization efficiencies and difficulties in standardization and quantification.^33,34^

Conversely, (semi) universal sensors, like total organic carbon (TOC) analyzers, charged aerosol detectors (CAD), and evaporative light-scattering detectors (ELSD), offer promise due to their carbon- (in the case of TOC) or mass-dependent (CAD and ELSD) response, independent of the analyte’s spectral or physicochemical properties. Their response is, however, significantly influenced by the mobile-phase composition,^35^ necessitating either complete removal of organic solvents after chromatography (e.g., TOC) or the use of inverse gradient compensation (e.g., CAD and ELSD).^36^ In fact, attempts have been made to use ELSD as a quantitative method for low molecular weight dissolved organic matter in natural waters.^37^ Nonetheless, its sensitivity to materials present in varying proportions within the sample renders its response only qualitatively useful for assessing the bulk chemical properties of a sample, as noted by others.^38^ Therefore, the development of a robust, simple, and inexpensive detection technique capable of quantifying the entirety of the matrix online during gradient HPLC purification would enhance the selectivity of the separation process straightforwardly.

One promising detector that could be used for this purpose is the quartz crystal microbalance (QCM). The QCM measures small mass changes with a subnanogram resolution on the surface of its oscillating piezoelectric quartz crystal by measuring changes in the oscillating resonance frequency as a function of deposited mass on its surface.^39,40^ Several studies showed how the QCM can be used to measure the sorption of the matrix NOM directly in a solution or the adsorption of dissolved compounds onto NOM and thus get insights into adsorption, adlayer formation, and interfacial dynamics of this matrix.^41−54^ NOM real-time quantification directly in liquid phase is, however, challenging to achieve due to (i) the limited capacity of available sensor surface coatings,^42^ (ii) the dependency of the sorption behavior on the type or fraction of NOM,^41,43^ the pH,^43,45^ and the continuous desorption,^45^ (iii) the often slow deposition rate,^42^ (iv) and other known challenges of liquid-based QCM measurements (e.g., viscous damping).^39,55^ The challenges of liquid-based QCM measurements can, however, be overcome using QCM dry-mass sensing introduced in the 1970s by Schulz and King.^56^ Technical advancement in the QCM and substantial optimization measures in the past decade makes dry-mass sensing seem to be an ideal solution for a robust and inexpensive strategy to monitor and quantify the entire matrix.^55,57−59^ In QCM dry-mass sensing, a small fraction of the HPLC column effluent is diverted and nebulized into micrometer-sized droplets using a microfluidic spray nozzle and sprayed onto the QCM sensor. The nebulized solvent evaporates, while nonvolatile components are deposited evenly on the QCM sensor, which can be quantified using the direct correlation between frequency change and mass.^55,58,59^ Kartanas et al.^59^ showed how this QCM dry-mass sensing could be used in combination with aquatic size-exclusion LC to separate and detect different proteins. It has, however, never been explored for a mixture as complex as an environmental extract. Moreover, the transition from aquatic to RP gradient elution is expected to cause variations in the QCM response as a result of changing fluid dynamics and evaporation rates. To this end, comprehensive characterization and validation of such a system are warranted to deal with organic solvents along with the development of a suitable calibration strategy.

The work presented in this and the companion paper^60^ has the overall goal of exploring the feasibility of coupling a commercial HPLC with a microfluidic spray dryer and a QCM for online monitoring of organic matrix components during RP HPLC gradient purification for mass spectrometry-based applications in environmental sciences. Both studies focus on organic matrices in already extracted samples, where most inorganic salts are excluded through a first solid-phase extraction step. The specific objectives of this paper were to (i) connect, characterize, and calibrate a microfluidic spray dryer with RP HPLC using an adjustable post-column flow splitter, (ii) define the lower and upper limits of quantification (LOQ) for QCM dry-mass sensing, and (iii) validate the online approach against offline TOC fraction analysis of NOM.

## Experimental Section

### Chemicals and Materials

A description of purchased chemicals, materials, and standard solutions used in this study is provided in the Supporting Information (Section S1).

### Instrumental Setup of QCM Dry-Mass Sensing

#### Coupling of QCM Dry-Mass Sensing with RP HPLC Using a Flow Splitter

**Figure 1:**
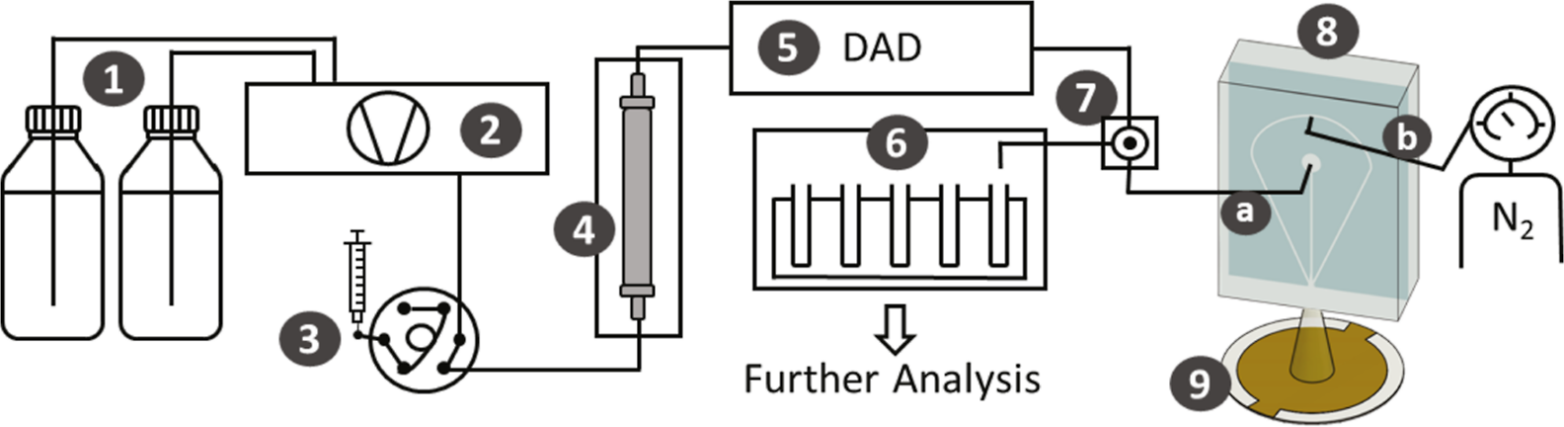
Schematic overview of the coupled HPLC–QCM system. (1) HPLC-grade solvents, (2) binary HPLC pump, (3) sample injector/autosampler, (4) chromatographic column; (5) DAD detector, (6) fraction collector, (7) post-column adjustable flow splitter, (8) microfluidic spray dryer, (a) connection to liquid channel, (b) connection to gas channel, and (9) QCM sensor.

An HPLC system, Figure 1 (parts 1–6), was coupled through an adjustable flow splitter, Figure 1(7), to QCM dry-mass sensing, Figure 1(8,9). Chromatography was performed on a Nexera XR HPLC system (Shimadzu, Japan) equipped with a solvent delivery module (LC-20AD, Shimadzu, Japan) (**2**), an RP column (**4**) (XTerra RP18 Column, length: 150 mm, diameter: 3.0 mm, particle size: 3.5 μ m, Waters, USA), a diode array detector (DAD) (**5**) (SPD-M20A, Shimadzu, Japan), and a fraction collector (**6**) (FRC-10A, Shimadzu, Japan). A flow rate of 0.5 mL/min, a sample injection volume of 200 μ L (**3**), and a column oven temperature of 40 °C were used for all HPLC measurements. Binary phase gradients with H_2_O (A) and 90% CH_3_OH and 10% H_2_O (B) were used as eluents (**1**).

The column effluent is split after the DAD using an analytical post-column adjustable flow splitter (**7**) (ASI 610-PO10-01, Analytical Scientific Instruments, USA, 50:1 to 1000:1 Split Ratio). Vernier scale settings were set to 65 (dimensionless) unless otherwise stated. The high-flow port was connected with the fraction collector (**6**) and the low-flow port with a microfluidic spray dryer (**8**) using polytetrafluorethylene (PTFE) tubing. The low-flow port was connected with the liquid channel of the spray dryer via PTFE tubes (**a**) (tube 1: outer diameter 1/16 in., inner diameter 0.010 in.; tube 2: outer diameter 1/32 in., inner diameter 1/75 in.) that were connected through an adapter (1/16 in. to 1/32 in., PEEK, IDEX Health and Science) to meet the requirements of both the splitter and the spray dryer.

We fabricated the microfluidic spray dryer (**8**) in-house using an optimized protocol (details in S1) of a previously published standard polydimethylsiloxane (PDMS) soft-lithography approach.^58^ The microfluidic spray dryer had two inlets: one connected to a nitrogen supply (**b**) set to 3 bar and another to the low-flow port of the flow splitter (**a**). The liquid channel had a length of 8.1 mm and a cross-section of 25 times 20 μm^2^, and the gas channels had a length of 8.4 mm and a cross-section of 100 times 70 μm^2^. The mobile phase was sprayed onto the frequency counter QCM200 from Stanford Research Systems (USA) equipped with a 5 MHz QCM crystal (**9**) (Stanford Research Systems 100RX1, Cr/Au, USA); a gate time of 0.1 s was used. To this end, the spray dryer was centered 3.5 cm above the QCM.

#### Determination of Split Ratios

Split ratios (*R*_split_) were determined for different Vernier scale settings (56, 66, 73, 79, 94, and 112) for three different CH_3_OH/H_2_O mobile phase compositions [85/15, 50/50, and 15/85 (v/v)] by spraying the mobile phase containing 500 mg/L NaCl for 30 min into a vial. The dried salt was reconstituted in 8 mL of H_2_O and the salt concentration in the solution was determined by measuring the salinity using a salinometer (MultiLine F/SET-3, WTW, Germany, see calculations in Section S2).

#### Measurement, Calibration, and Data Processing

Prior to each sample, a blank was run on the system described in Figure 1 under identical conditions where 200 μL of 25/75 CH_3_OH/H_2_O (v/v) was injected instead of the sample. After the sample measurement, a one-point calibration was performed through constant mass spraying on the sensor achieved under the same chromatographic conditions but with the eluents containing NaCl (*c*_cal_ = 300 mg/L). The obtained frequencies given in Hz for each time point were translated into mass concentrations given in milligrams per liter using eq 1, as well as detailed in a Matlab script (see Section S6).
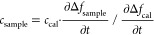
1In this procedure, the blank (*f*_blank_) is subtracted from both the sample (*f*_sample_) and the calibration measurement (*f*_cal_) to obtain corrected frequencies Δ*f*_sample_ and Δ*f*_cal_, respectively. Then, the first derivative (∂Δ*f*_sample_/∂*t* and ∂Δ*f*_cal_/∂*t*) is produced and smoothed using a Savitzky–Golay filter (polynomial order 3, 301 points). The derivative ratio multiplied by the salt concentration for calibration (*c*_cal_) yields mass concentrations in the sample (*c*_sample_).

### Determination of Lower and Upper Limits of Detection and Quantification

The limits of detection (LOD) and quantification (LOQ) of the system were determined for three different CH_3_OH/H_2_O mobile phase compositions [85/15, 50/50, and 15/85 (v/v)] according to the calibration method (DIN 32645).^61^ To this end, we sprayed the mobile phase containing NaCl in different concentrations (0, 30, 60, 90, 120, 150, and 180 mg/L) for 10 min onto the QCM. The average slope of quadruplicates of the frequency decrease was used for the signal intensity.

For the determination of the upper limit of the system for the three different mobile phase compositions, the mobile phase containing 500 mg/L NaCl was sprayed onto the QCM in triplicates. The upper limit was then estimated by calculating the amount of salt being sprayed until the point when the energy loss in the system, which was determined using the motional resistance, reached a critical point, namely, where the resistance was by *a* factor of 3 higher than the starting resistance (12–17 Ω), but the noise change over time was still ≤5 Hz/min and a difference of the frequency change ≤7 Hz/min among triplicates.

### Validation of the QCM Dry-Mass Sensing Approach

We compared dry-mass sensing with a TOC analysis to validate our measurement approach. The elution of 1.65 mg NOM during a typical HPLC gradient (see Table S2) was monitored and quantified online using QCM dry-mass sensing (gate time: 0.1 s) and offline using TOC analysis. To this end, fractions of the HPLC eluate were taken every 30 s by using a fraction collector (**6** in Figure 1). The fractionated eluate was evaporated to dryness under a gentle stream of N_2_ at 30 °C and then reconstituted in 16 mL of H_2_O. Organic carbon concentrations in each reconstituted sample were determined using a TOC analyzer (TOC-L, Shimadzu, Japan) equipped with a combustion catalytic oxidation reactor (680 °C) and a nondispersive infrared detector to analyze the generated CO_2_.

For extraction of riverine NOM, samples were taken from the creek Wiesäckerbach (Garching, Germany, latitude 48.269009, longitude 11.667976) and filtered through glass microfiber filter membranes (1.2 μm particle retention, 47 mm diameter, Whatman, UK). The filtered samples were passed over OASIS HLB cartridges under conventional solid-phase extraction conditions (Waters, 200 mg, 6 cc) using an automated SPE system (Smart Prep Extractor, Horizon Technology, USA) at 5 mL/min. The cartridges were subsequently dried under vacuum overnight and eluted in 5 mL of CH_3_OH. The volume of combined eluates was reduced under a gentle stream of N_2_ at 30 °C and then stored at −18 °C.

## Results and Discussion

### Coupling, Flow Control, and Calibration

**Figure 2:**
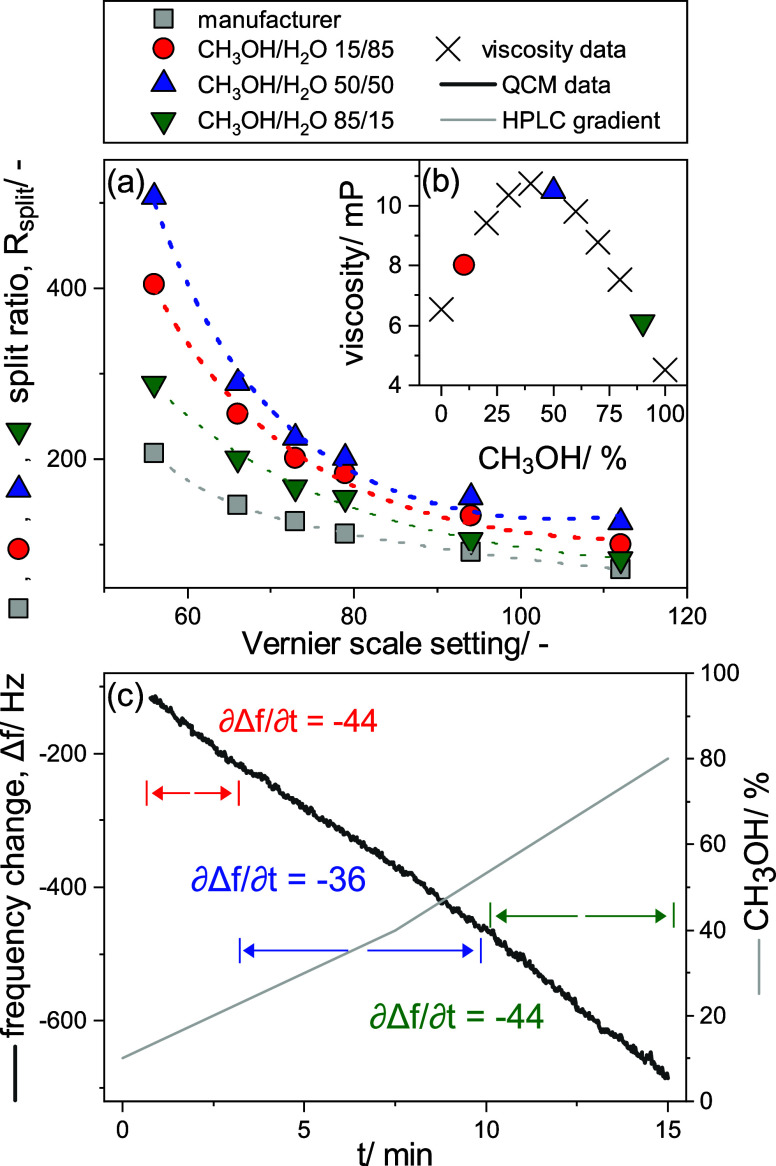
(a) Comparison of split ratios measured for the HPLC–QCM system at different Vernier scale settings for three different CH_3_OH/H_2_O compositions at 40 °C. The values were fitted with an exponential decay function. The values provided by the manufacturer at 25 °C for pure H_2_O are shown in gray. (b) Viscosity values of CH_3_OH/H_2_O mixtures at 40 °C from Mikhail and Kimel^62^ in millipoise (mP). (c) Nonlinear response of constantly spraying 300 mg/L NaCl as a function of eluent composition. The frequency shift is shown in black, whereas the eluent composition during the gradient run is shown in gray in vol % of CH_3_OH. Three different operational regions and the respective linear regression were defined according to the % of CH_3_OH in the mobile phase and the corresponding viscosity.

We coupled an HPLC system (**1**–**6** in Figure 1, flow range = 0.1–1 mL/min) with a microfluidic spray dryer and QCM sensor (**8** and **9**, respectively; flow range = 1–4 μL/min) through an adjustable flow splitter (**7**). The narrow flow dynamic range of components **8** and **9** warrants accurate control of the split ratio under chromatographic conditions. Additionally, the changing viscosity as a result of gradient HPLC is expected to have an influence on the split ratios and on the spray behavior. Therefore, we evaluated split ratios for three different solvent compositions (CH_3_OH/H_2_O 15/85, 50/50, and 85/15 v/v) and six different Vernier scale settings in the range between 56 and 112 (see Figure 2a).

The split ratios increase up to 2.4 times for the HPLC–QCM dry-mass sensing system using CH_3_OH/H_2_O solvent compositions (Figure 2a blue, red, and green data) in comparison with the data provided by the manufacturer (Figure 2a, gray data), using only H_2_O as a solvent with no restrictions after the flow splitter. Moreover, variations by *a* factor of 1.1–1.8 were observed for different solvent compositions in the order CH_3_OH/H_2_O 50/50 (blue) > 15/85 (red) > 85/15 (green). These variations are significant enough to induce shifts toward lower flow rates to the spray dryer that may lead to operation outside its dynamic range (split ratios between 125 and 500), thus affirming the need to quantify split ratios for the system. Although entrapment of residual solvent(s) within the deposited mass on QCM could be suspected as a possible reason for the varying response, we excluded this possibility as the frequency shift immediately stabilized and stayed constant upon a longer drying period regardless of the eluent composition (see Supporting Information, Section S3 and Figure S1). In fact, the overall split ratio shift for the HPLC–QCM dry-mass sensing system is caused by backpressure originating from the small inner diameter of the liquid channel of the spray dryer (25 × 20 μm^2^), which is the only additional constriction in the system compared to manufacturer’s data.^63−65^ In addition, the pressure in the system leads to a deformation of the liquid channel made of PDMS and thus to an increase of its hydraulic diameter (*D*_hyd_) as reported by Kartanas et al.^59^ This deformation strongly influences the hydraulic resistance [*R*_res_ ∝ 1/(*D*_hyd_)^4^] and thus the generated backpressure. Since *D*_hyd_ is dependent on the liquid dynamic viscosity, the trend of the split ratios must follow the trend of the dynamic viscosities of the respective solvent compositions. Indeed, this is the case as shown in Figure 2b where the highest viscosity is for CH_3_OH/H_2_O 50/50 (10.5 mP, blue) > 10/90 (8.0 mP, red) > 90/10 (6.1 mP, green).

These results imply that while the split ratio, and thus the flow to the microfluidic spray dryer, will stay constant during an isocratic HPLC run, flows will constantly change in gradient HPLC mode. If the split ratio shifts are within the dynamic range (split ratios between 125 and 500), the QCM dry-mass sensing response is still expected to vary. Indeed, spraying a fixed concentration of solute (300 mg/L NaCl) returns a nonlinear frequency response (Figure 2c) during the CH_3_OH/H_2_O gradient run (Figure 2c, gray). The slope of the frequency change as a function of CH_3_OH content follows in fact the observed shifts in split ratio earlier determined in the order CH_3_OH 25–55% (∂Δ*f*/∂*t*: −36, blue) < 10–25% and 55–90% (∂Δ*f*/∂*t*: −44, red and green). While this observation follows the viscosity regions, it is conceivably not only a result of the split ratio shifts during the gradient run but a combination of split ratio changes and changes in the spraying behavior. Based on these results, we developed a calibration and data processing strategy, where we prepare calibration solvents (e.g., H_2_O, CH_3_OH) with a NaCl concentration of 300 mg/L for binary solvent systems and record the QCM response as a function of time during the same gradient run of the sample. This calibration is used to quantify the amount of solutes in the sample by dividing the frequency change of the sample by one of the calibrations. This strategy is valid not only for solute concentrations of 300 mg/L but also for a large range of concentrations as the sprayed mass gives a linear response of the frequency change in this range (30–500 mg/L; see the Supporting Information in Figure S3).

### Evaluation of Lower and Upper Quantification Limits

The concentration range in which accurate quantification of masses is achievable using the presented QCM dry-mass sensing approach was investigated next by spraying different concentrations of NaCl (from 0 to 180 mg/L) in a binary mobile phase system (i.e., CH_3_OH/H_2_O) for three different compositions [CH_3_OH/H_2_O 15/85, 50/50, and 85/15 (v/v)]. The LOD and LOQ were determined according to the calibration method by using the slope of the frequency change per minute for each measurement as the signal intensity term (y); the results are shown in Table 1.

**Table 1:** LODs, LOQs, and Upper Limit Determined for Dry-Mass Sensing for Three Solvent Compositions over a 10 min Duration

			upper limit	
CH_3_OH/H_2_O (v/v)	LOD (mg/L)	LOQ (mg/L)	mg/L	μg
15/85	15	52	336 ± 7	6.6 ± 0.1
50/50	4.3	16	408 ± 2	7.1 ± 0.1
85/15	12	42	475 ± 4	11.8 ± 0.1

The LOD for the different mobile phase compositions ranged from 4.3 to 15 mg/L, whereas the LOQ was found to be from 16 to 52 mg/L. The presented system’s detection limits are at least 6 times lower than that estimated by Kartanas et al.^59^ for aquatic conditions (LOQ: 100 mg/L). Although we observed lower noise levels in this study (5–15 Hz) compared with Kartanas et al.^59^ (aquatic: 30 Hz), this cannot alone explain these results. In our study, the lowest detection limits were determined for CH_3_OH/H_2_O 50/50 composition (LOD = 4.3 mg/L, noise = 10 Hz), whereas the observed noise was found to be the smallest for CH_3_OH/H_2_O 85/15 composition (LOD = 12 mg/L, noise = 5 Hz). Flow rates cannot fully explain the observed trends of the detection limits either, as they do not follow the same order we observed earlier. This indicates that the exact limits are not merely dependent on the noise and the flow rate, but also on other interconnected factors that are influenced by both the solvent composition and the flow rate including the spray cone dimensions, uniformity of the generated spray, droplet size, and evaporation rate of solvent(s).^66−68^

There exists, however, not only a lower limit of the QCM quantification but also an upper limit, above which both the trueness and precision of QCM dry-mass sensing are compromised. Indeed, spraying a salt solution with a high concentration (500 mg/L) on the QCM led over time to an increase in noise, to a shift of the slope of the frequency change, and to increased resistance values measured on the QCM (see Figure S2). Since higher resistance values indicate a change in the oscillation behavior of the quartz crystal, and hence a change of the frequency–mass correlation,^69^ we defined an operational upper limit when the measured motional resistance is by *a* factor of 3 higher than the starting resistance. This corresponds to a change of the noise over time ≤5 Hz/min and a difference of the frequency change ≤7 Hz/min among triplicates. The upper limit was found to be above 6.6 μg salt sprayed onto the sensor for all solvent compositions, which corresponds for a 10 min measurement to an upper concentration limit of above 330 mg/L (see Table 1), which corresponds to 2 measurements at *c*_sample_ = 5000 mg/L (injection volume = 200 μL) and *R*_split_ = 200–290. This suggests that cleaning the QCM sensor after each measurement may be necessary to guarantee reproducible and accurate results. Such a step was accomplished in this study by 2–3 gentle swipes of the QCM sensor surface using a wet microfiber cloth that proved to be effective with no significant deviation of frequency change over time even after 100 deposition and cleaning cycles (deviation from the new sensor: ∂Δ*f*/∂*t* = 0.4 ± 2.4 Hz/min). Alternatively, depositing a droplet of CH_3_OH/H_2_O on the surface and blowing it away using pressurized air was equally effective—a step that can be easily automated.

### Validation of Online QCM Dry-Mass Sensing against Offline TOC Fraction Analysis for Organic Matrix

**Figure 3:**
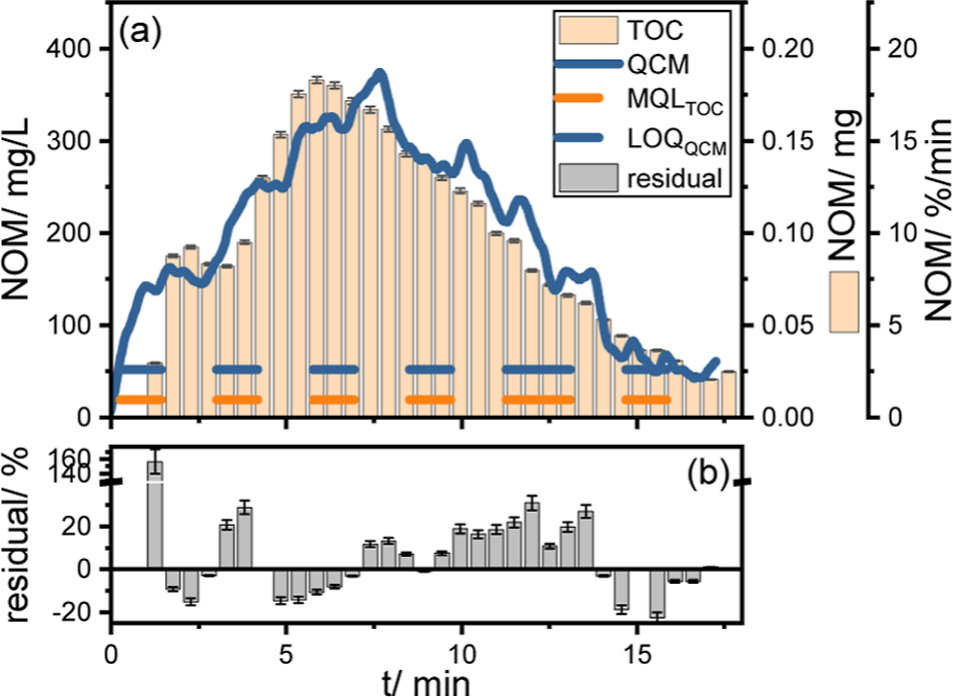
(a) Comparison of the NOM matrix monitoring results obtained by dry-mass sensing (blue line, one measurement point every 0.1 s) with the results of offline TOC analysis of LC fractions (orange bar chart, one fraction every 30 s) during the isocratic HPLC separation (CH_3_OH/H_2_O 30/70 v/v; 0.5 mL/min) of NOM (injected mass = 1 mg) on column (XTerra RP18, 150 × 3.0 mm, 3.5 μ m). Orange dashed line: MQL of TOC analysis (13.2 mg/L). Blue dashed line: LOQ of QCM (40 mg/L). (b) Gray bar chart: variance of QCM and TOC measurement per fraction shown as residual in %.

We validated the accuracy of QCM dry-mass sensing coupled to HPLC by real-time monitoring of a NOM extract during HPLC separation using our system and compared it with the results of offline TOC analysis of collected HPLC fractions during the same run as a reference strategy. This was possible since the extracted NOM could be reconstituted in H_2_O without significant losses (NOM recovery ≥98%), while inorganic salts were already removed during the pre-extraction step. NOM quantification using QCM dry-mass sensing (see Figure 3a, blue line, see Figure S5 for QCM raw data) is in good agreement with fraction analysis using TOC (Figure 3a, orange bars). Both detection techniques show that NOM elutes as an unresolved hump with maxima between 4 and 9 min, which is a typical behavior of NOM during RP HPLC using C18 separation columns of similar dimensions.^70,71^ Moreover, absolute recoveries obtained by real-time QCM dry-mass sensing (=103 ± 10%) agree well with those of offline TOC fraction analysis (=98 ± 1%). These results confirm the suitability of QCM dry-mass sensing for the real-time monitoring of complex matrices during a typical HPLC separation. The lower precision achieved by QCM dry-mass sensing (=± 10%) compared to TOC analysis (=± 1%) is conceivably the result of the variations associated with the continuous spraying/evaporation processes (typical precision of QCM measurements without spray drying: 0.1 Hz).^57^ Yet, these results are significantly more precise than ELSD, which could only achieve precisions ≥ ± 30% for NOM.^37^

Analysis of variance shows a slight tendency of the QCM to overestimate the NOM (see Figure 3b). This overestimation sums up to a total of 5 ± 11% until the retention time where the LOQ for QCM is reached (*t* = 17.2 min, LOQ_QCM_ = 52 mg/L, Figure 3a blue dashed line), which is still within the measured QCM precision. Note that the LOQ of dry-mass sensing is 2.7 times higher than the method quantification limit of offline TOC analysis (MQL = 19.2 mg/L, Figure 3a orange dashed line, for calculation from LOD to MQL see Figure S4). This narrow sensitivity gap could easily be closed by further fine-tuning the performance of the spray dryer, such as spraying a higher fraction of the mass in the mobile phase on the QCM.

## Conclusions

The current work presents the successful coupling of a commercial HPLC system with a QCM using a microfluidic spray dryer. We demonstrate that QCM dry-mass sensing is suitable as a holistic detector for quantifying complex organic matrix during HPLC cleanup. Validation against offline TOC analysis confirmed the successful coupling, calibration, and data processing strategy and its suitability within a precision of 10%. Furthermore, the current limit of QCM dry-mass sensing (LOQ = 16–52 mg/L) is in a comparable range as other (semi-) universal detectors (TOC, MLQ = 19.2 mg/L; ELSD, LOQ = 80 mg/L^37^), but with the powerful advantage of using gradient solvents without the need for solvent removal or compensation. While QCM dry-mass sensing was successful in online quantification of the sample matrix, it is unspecific to target analytes. This makes it useful when the latter does not constitute a significant proportion of the total mass in the sample (e.g., ≤1%). Nonetheless, combining a selective detector, such as UV–vis, and QCM dry-mass sensing will yield complete data on both matrix and target analytes and thus enable the optimization of HPLC cleanup procedures. This approach can possibly be further extended to other fields (e.g., food, archeology, and forensics) with similar loads of matrix, whereas further reduction of the system LOQ may be necessary for more matrix-susceptible samples. In conclusion, the developed system can be a useful tool to minimize matrix coelution, thereby reducing adverse matrix effects in a subsequent analysis, which is the ultimate goal of a cleanup. The exact potential gain of such an optimized cleanup is further evaluated in the companion paper.^60^

## Supporting Information Available

Further information on chemicals and materials, additional experimental details and results, and MATLAB code for data processing (PDF)

## References

[ref1] Nasiri M.; Ahmadzadeh H.; Amiri A. Sample preparation and extraction methods for pesticides in aquatic environments: A review. Trends Anal. Chem. 2020, 123, 115772 10.1016/j.trac.2019.115772.

[ref2] Elsner M.; Imfeld G. Compound-specific isotope analysis (CSIA) of micropollutants in the environment - current developments and future challenges. Curr. Opin. Biotechnol. 2016, 41, 60–72. 10.1016/j.copbio.2016.04.014. 27340797

[ref3] Tassi M.; De Vos J.; Chatterjee S.; Sobott F.; Bones J.; Eeltink S. Advances in native high-performance liquid chromatography and intact mass spectrometry for the characterization of biopharmaceutical products. J. Sep. Sci. 2018, 41, 125–144. 10.1002/jssc.201700988. 28990739

[ref4] Xie Z.; Feng Q.; Zhang S.; Yan Y.; Deng C.; Ding C. Advances in proteomics sample preparation and enrichment for phosphorylation and glycosylation analysis. Proteomics 2022, 22, e2200070 10.1002/pmic.202200070. 36100958

[ref5] Beyer A.; Biziuk M. K. Applications of sample preparation techniques in the analysis of pesticides and PCBs in food. Food Chem. 2008, 108, 669–680. 10.1016/j.foodchem.2007.11.024. 26059147

[ref6] Lambropoulou D. A.; Albanis T. A. Methods of sample preparation for determination of pesticide residues in food matrices by chromatography–mass spectrometry-based techniques: a review. Anal. Bioanal. Chem. 2007, 389, 1663–1683. 10.1007/s00216-007-1348-2. 17541563

[ref7] Zhang L.; Liu S.; Cui X.; Pan C.; Zhang A.; Chen F. A review of sample preparation methods for the pesticide residue analysis in foods. Cent. Eur. J. Chem. 2012, 10, 900–925. 10.2478/s11532-012-0034-1.

[ref8] Hajšlová J.; Zrostlíková J. Matrix effects in (ultra)trace analysis of pesticide residues in food and biotic matrices. J. Chromatogr. A 2003, 1000, 181–197. 10.1016/S0021-9673(03)00539-9. 12877171

[ref9] Rapp-Wright H.; McEneff G. L.; Murphy B.; Gamble S.; Morgan R. M.; Beardah M. S.; Barron L. P. Suspect screening and quantification of trace organic explosives in wastewater using solid phase extraction and liquid chromatography-high resolution accurate mass spectrometry. J. Hazard. Mater. 2017, 329, 11–21. 10.1016/j.jhazmat.2017.01.008. 28119193

[ref10] Ridgway K.; Lalljie S. P.; Smith R. M. Sample preparation techniques for the determination of trace residues and contaminants in foods. J. Chromatogr. A 2007, 1153, 36–53. 10.1016/j.chroma.2007.01.134. 17313955

[ref11] Angeles L. F.; Aga D. S. Catching the elusive persistent and mobile organic compounds: Novel sample preparation and advanced analytical techniques. Trends Environ. Anal. Chem. 2020, 25, e00078 10.1016/j.teac.2019.e00078.

[ref12] Knoll S.; Rösch T.; Huhn C. Trends in sample preparation and separation methods for the analysis of very polar and ionic compounds in environmental water and biota samples. Anal. Bioanal. Chem. 2020, 412, 6149–6165. 10.1007/s00216-020-02811-5. 32710277 PMC7442764

[ref13] da Silva Burato J. S.; Medina D. A. V.; de Toffoli A. L.; Maciel E. V. S.; Lanças F. M. Recent advances and trends in miniaturized sample preparation techniques. J. Sep. Sci. 2019, 43, 202–225. 10.1002/jssc.201900776. 31692234

[ref14] Płotka-Wasylka J.; Szczepańska N.; de la Guardia M.; Namieśnik J. Miniaturized solid-phase extraction techniques. Trends Anal. Chem. 2015, 73, 19–38. 10.1016/j.trac.2015.04.026.

[ref15] Ribeiro C. M. R.; Ribeiro A. R. L.; Maia A. S.; Gonçalves V. M. F.; Tiritan M. E. New Trends in Sample Preparation Techniques for Environmental Analysis. Crit. Rev. Anal. Chem. 2014, 44, 142–185. 10.1080/10408347.2013.833850. 25391434

[ref16] Moser A. C.; Hage D. S. Immunoaffinity chromatography: an introduction to applications and recent developments. Bioanalysis 2010, 2, 769–790. 10.4155/bio.10.31. 20640220 PMC2903764

[ref17] Gu Z.; Yang C.; Chang N.; Yan X. Metal-organic frameworks for analytical chemistry: from sample collection to chromatographic separation. Acc. Chem. Res. 2012, 45, 734–745. 10.1021/ar2002599. 22404189

[ref18] Thurman E. M. Developments in Biogeochemistry; Springer, 1985.

[ref19] Sugitate K.; Nakamura S.; Orikata N.; Mizukoshi K.; Nakamura M.; Toriba A.; Hayakawa K. Search of components causing matrix effects on GC/MS for pesticide analysis in food. J. Pestic. Sci. 2012, 37, 156–163. 10.1584/jpestics.D11-048.

[ref20] Her N.; Amy G.; McKnight D. M.; Sohn J.; Yoon Y. Characterization of DOM as a function of MW by fluorescence EEM and HPLC-SEC using UVA, DOC, and fluorescence detection. Water Res. 2003, 37, 4295–4303. 10.1016/S0043-1354(03)00317-8. 12946913

[ref21] Liu R.; Lead J. R.; Baker A. Fluorescence characterization of cross flow ultrafiltration derived freshwater colloidal and dissolved organic matter. Chemosphere 2007, 68, 1304–1311. 10.1016/j.chemosphere.2007.01.048. 17350076

[ref22] Seredyńska-Sobecka B.; Baker A.; Lead J. R. Characterisation of colloidal and particulate organic carbon in freshwaters by thermal fluorescence quenching. Water Res. 2007, 41, 3069–3076. 10.1016/j.watres.2007.04.017. 17560624

[ref23] Bieroza M.; Baker A.; Bridgeman J. Relating freshwater organic matter fluorescence to organic carbon removal efficiency in drinking water treatment. Sci. Total Environ. 2009, 407, 1765–1774. 10.1016/j.scitotenv.2008.11.013. 19081606

[ref24] Baghoth S.; Sharma S.; Amy G. Tracking natural organic matter (NOM) in a drinking water treatment plant using fluorescence excitation–emission matrices and PARAFAC. Water Res. 2011, 45, 797–809. 10.1016/j.watres.2010.09.005. 20889181

[ref25] Kim H.-C.; Yu M.-J. Characterization of aquatic humic substances to DBPs formation in advanced treatment processes for conventionally treated water. J. Hazard. Mater. 2007, 143, 486–493. 10.1016/j.jhazmat.2006.09.063. 17092645

[ref26] Helms J. R.; Stubbins A.; Ritchie J. D.; Minor E. C.; Kieber D. J.; Mopper K. Absorption spectral slopes and slope ratios as indicators of molecular weight, source, and photobleaching of chromophoric dissolved organic matter. Limnol. Oceanogr. 2008, 53, 955–969. 10.4319/lo.2008.53.3.0955.

[ref27] Roccaro P.; Yan M.; Korshin G. V. Use of log-transformed absorbance spectra for online monitoring of the reactivity of natural organic matter. Water Res. 2015, 84, 136–143. 10.1016/j.watres.2015.07.029. 26231579

[ref28] Li P.; Hur J. Utilization of UV-Vis spectroscopy and related data analyses for dissolved organic matter (DOM) studies: A review. Crit. Rev. Environ. Sci. Technol. 2017, 47, 131–154. 10.1080/10643389.2017.1309186.

[ref29] Matilainen A.; Gjessing E. T.; Lahtinen T.; Hed L.; Bhatnagar A.; Sillanpää M. An overview of the methods used in the characterisation of natural organic matter (NOM) in relation to drinking water treatment. Chemosphere 2011, 83, 1431–1442. 10.1016/j.chemosphere.2011.01.018. 21316073

[ref30] Chen W.; Yu H.-Q. Advances in the characterization and monitoring of natural organic matter using spectroscopic approaches. Water Res. 2021, 190, 116759 10.1016/j.watres.2020.116759. 33360618

[ref31] Spencer R. G.; Bolton L.; Baker A. Freeze/thaw and pH effects on freshwater dissolved organic matter fluorescence and absorbance properties from a number of UK locations. Water Res. 2007, 41, 2941–2950. 10.1016/j.watres.2007.04.012. 17540432

[ref32] Groeneveld M.; Catalán N.; Einarsdottir K.; Bravo A. G.; Kothawala D. N. The influence of pH on dissolved organic matter fluorescence in inland waters. Anal. Methods 2022, 14, 1351–1360. 10.1039/D1AY01702K. 35298579

[ref33] Patriarca C.; Balderrama A.; Može M.; Sjöberg P. J. R.; Bergquist J.; Tranvik L. J.; Hawkes J. A. Investigating the Ionization of Dissolved Organic Matter by Electrospray. Anal. Chem. 2020, 92, 14210–14218. 10.1021/acs.analchem.0c03438. 32940031 PMC7584329

[ref34] Hawkes J. A.; D’Andrilli J.; Agar J. N.; Barrow M. P.; Berg S. M.; Catalán N.; Chen H.; Chu R. K.; Cole R. B.; Dittmar T.; et al. An international laboratory comparison of dissolved organic matter composition by high resolution mass spectrometry: Are we getting the same answer?. Limnol Oceanogr. Methods 2020, 18, 235–258. 10.1002/lom3.10364.

[ref35] Vehovec T.; Obreza A. Review of operating principle and applications of the charged aerosol detector. J. Chromatogr. A 2010, 1217, 1549–1556. 10.1016/j.chroma.2010.01.007. 20083252

[ref36] de Villiers A.; Górecki T.; Lynen F.; Szucs R.; Sandra P. A. T. Improving the universal response of evaporative light scattering detection by mobile phase compensation. J. Chromatogr. A 2007, 1161, 183–191. 10.1016/j.chroma.2007.05.078. 17568599

[ref37] Rojas A.; Sandron S.; Wilson R.; Davies N. W.; Haddad P. R.; Shellie R. A.; Nesterenko P. N.; Paull B. Simple, quantitative method for low molecular weight dissolved organic matter extracted from natural waters based upon high performance counter-current chromatography. Anal. Chim. Acta 2016, 909, 129–138. 10.1016/j.aca.2016.01.003. 26851093

[ref38] Acworth I. N.; Thomas D. Charged aerosol detection and evaporative light scattering detection – Fundamental differences affecting analytical performance. Planta Med. 2014, 80, PPL2 10.1055/s-0034-1382638.

[ref39] Reviakine I.; Johannsmann D.; Richter R. P. Hearing what you cannot see and visualizing what you hear: interpreting quartz crystal microbalance data from solvated interfaces. Anal. Chem. 2011, 83, 8838–8848. 10.1021/ac201778h. 21939220

[ref40] Sauerbrey G. Verwendung von Schwingquarzen zur Wägung dünner Schichten und zur Mikrowägung. Z. Phys. 1959, 155, 206–222. 10.1007/BF01337937.

[ref41] Armanious A.; Aeppli M.; Sander M. Dissolved organic matter adsorption to model surfaces: adlayer formation, properties, and dynamics at the nanoscale. Environ. Sci. Technol. 2014, 48, 9420–9429. 10.1021/es5026917. 25024044

[ref42] Li W.; Liao P.; Oldham T.; Jiang Y.; Pan C.; Yuan S.; Fortner J. D. Real-time evaluation of natural organic matter deposition processes onto model environmental surfaces. Water Res. 2018, 129, 231–239. 10.1016/j.watres.2017.11.024. 29153876

[ref43] Yan M.; Liu C.; Wang D.; Ni J.; Cheng J. Characterization of adsorption of humic acid onto alumina using quartz crystal microbalance with dissipation. Langmuir 2011, 27, 9860–9865. 10.1021/la1042102. 21774560

[ref44] Wang X.; Huang D.; Cheng B.; Wang L. New insight into the adsorption behaviour of effluent organic matter on organic–inorganic ultrafiltration membranes: a combined QCM-D and AFM study. R. Soc. Open Sci. 2018, 5, 180586 10.1098/rsos.180586. 30225052 PMC6124109

[ref45] Eita M. In situ study of the adsorption of humic acid on the surface of aluminium oxide by QCM-D reveals novel features. Soft Matter 2011, 7, 709–715. 10.1039/C0SM00648C.

[ref46] Eita M. Insight into the adsorption of humic acid/Gd3+ complex on the surface of Al2O3 studied in situ by QCM-D and ex situ by ellipsometry and XPS. Soft Matter 2011, 7, 7424–7430. 10.1039/c1sm05806a.

[ref47] Yan M.; Wang D.; Xie J.; Liu C.; Cheng J.; Chow C. W. K.; van Leeuwen J. Investigation of the adsorption characteristics of natural organic matter from typical Chinese surface waters onto alumina using quartz crystal microbalance with dissipation. J. Hazard. Mater. 2012, 215–216, 115–121. 10.1016/j.jhazmat.2012.02.039. 22444034

[ref48] Zeng T.; Wilson C. J.; Mitch W. A. Effect of chemical oxidation on the sorption tendency of dissolved organic matter to a model hydrophobic surface. Environ. Sci. Technol. 2014, 48, 5118–5126. 10.1021/es405257b. 24697505

[ref49] Sander M.; Tomaszewski J. E.; Madliger M.; Schwarzenbach R. P. Adsorption of insecticidal Cry1Ab protein to humic substances. 1. Experimental approach and mechanistic aspects. Environ. Sci. Technol. 2012, 46, 9923–9931. 10.1021/es3022478. 22862304

[ref50] Tomaszewski J. E.; Madliger M.; Pedersen J. A.; Schwarzenbach R. P.; Sander M. Adsorption of insecticidal Cry1Ab protein to humic substances. 2. Influence of humic and fulvic acid charge and polarity characteristics. Environ. Sci. Technol. 2012, 46, 9932–9940. 10.1021/es302248u. 22862550

[ref51] Nguyen T. H.; Elimelech M. Adsorption of plasmid DNA to a natural organic matter-coated silica surface: kinetics, conformation, and reversibility. Langmuir 2007, 23, 3273–3279. 10.1021/la0622525. 17286415

[ref52] Nguyen T. H.; Chen K. L. Role of divalent cations in plasmid DNA adsorption to natural organic matter-coated silica surface. Environ. Sci. Technol. 2007, 41, 5370–5375. 10.1021/es070425m. 17822104

[ref53] Furman O.; Usenko S.; Lau B. L. T. Relative importance of the humic and fulvic fractions of natural organic matter in the aggregation and deposition of silver nanoparticles. Environ. Sci. Technol. 2013, 47, 1349–1356. 10.1021/es303275g. 23298221

[ref54] Tomaszewski J. E.; Schwarzenbach R. P.; Sander M. Protein encapsulation by humic substances. Environ. Sci. Technol. 2011, 45, 6003–6010. 10.1021/es200663h. 21678916

[ref55] Müller T.; White D. A.; Knowles T. P. J. Dry-Mass Sensing for Microfluidics. Appl. Phys. Lett. 2014, 105, 214101 10.1063/1.4902131.

[ref56] Schulz W. W.; King W. H. A Universal Mass Detector for Liquid Chromatography. J. Chromatogr. Sci. 1973, 11, 343–348. 10.1093/chromsci/11.7.343.

[ref57] Johannsmann D. The Quartz Crystal Microbalance in Soft Matter Research; Springer, 2015.

[ref58] Kartanas T.; Ostanin V. P.; Challa P. K.; Daly R.; Charmet J.; Knowles T. P. J. Enhanced Quality Factor Label-free Biosensing with Micro-Cantilevers Integrated into Microfluidic Systems. Anal. Chem. 2017, 89, 11929–11936. 10.1021/acs.analchem.7b01174. 28984439

[ref59] Kartanas T.; Levin A.; Toprakcioglu Z.; Scheidt T.; Hakala T. A.; Charmet J.; Knowles T. P. J. Label-Free Protein Analysis Using Liquid Chromatography with Gravimetric Detection. Anal. Chem. 2021, 93, 2848–2853. 10.1021/acs.analchem.0c04149. 33507064

[ref60] Wabnitz C.; Chen W.; Elsner M.; Bakkour R. Quartz Crystal Microbalance as Holistic Detector for Quantifying Complex Organic Matrices During Liquid Chromatography: 2. Compound Specific Isotope Analysis. Anal. Chem. 2023, 10.1021/acs.analchem.3c05441, under revision. PMC11099894 38700939

[ref61] DIN 32645: Chemical Analysis—Decision Limit, Detection Limit and Determination Limit under Repeatability Conditions—Terms, Methods; Evaluation; Beuth Verlag GmbH, 2008.

[ref62] Mikhail S. Z.; Kimel W. Densities and Viscosities of Methanol-Water Mixtures. J. Chem. Eng. Data 1961, 6, 533–537. 10.1021/je60011a015.

[ref63] https://www.hplc-asi.com/flow-splitters/(accessed Dec 02, 2022).

[ref64] Marks R. G. H.; Jochmann M. A.; Brand W. A.; Schmidt T. C. How to Couple LC-IRMS with HRMS - A Proof-of-Concept Study. Anal. Chem. 2022, 94, 2981–2987. 10.1021/acs.analchem.1c05226. 35107978

[ref65] Gunnarson C.; Lauer T.; Willenbring H.; Larson E. J.; Dittmann M.; Broeckhoven K.; Stoll D. R. Implications of dispersion in connecting capillaries for separation systems involving post-column flow splitting. J. Chromatogr. A 2021, 1639, 461893 10.1016/j.chroma.2021.461893. 33524933

[ref66] Kartanas T.; Rodrigues R.; Müller T.; Herling T. W.; Knowles T. P. J.; Charmet J. 3D microfluidics spray nozzle for sample processing and materials deposition. AIP Conference Proceedings; AIP Publishing, 2019; 2092.

[ref67] Kartanas T.; Toprakcioglu Z.; Hakala T. A.; Levin A.; Herling T. W.; Daly R.; Charmet J.; Knowles T. P. J. Mechanism of droplet-formation in a supersonic microfluidic spray device. Appl. Phys. Lett. 2020, 116, 153702 10.1063/1.5145109.

[ref68] Hu H.; Larson R. G. Evaporation of a Sessile Droplet on a Substrate. J. Phys. Chem. B 2002, 106, 1334–1344. 10.1021/jp0118322.

[ref69] Martin S. J.; Frye G. C.; Wessendorf K. O. Sensing liquid properties with thickness-shear mode resonators. Sens. Actuators, A 1994, 44, 209–218. 10.1016/0924-4247(94)00806-X.

[ref70] Sandron S.; Rojas A.; Wilson R.; Davies N. W.; Haddad P. R.; Shellie R. A.; Nesterenko P. N.; Kelleher B. P.; Paull B. Chromatographic methods for the isolation, separation and characterisation of dissolved organic matter. Environ. Sci.: Processes Impacts 2015, 17, 1531–1567. 10.1039/C5EM00223K. 26290053

[ref71] Wu F. C.; Evans R. D.; Dillon P. High-performance liquid chromatographic fractionation and characterization of fulvic acid. Anal. Chim. Acta 2002, 464, 47–55. 10.1016/S0003-2670(02)00476-2.

