# Supplementary material for: Quartz Crystal Microbalance as a Holistic Detector for Quantifying Complex Organic Matrices during Liquid Chromatography: 1. Coupling, Characterization, and Validation
Source: Anal Chem. 2024 Apr 29;96(19):7429–35. doi: 10.1021/acs.analchem.3c05440 (PMC11099895; doi:10.1021/acs.analchem.3c05440)
Supplement: Supplementary file 1 — ac3c05440_si_001.pdf [file ac3c05440_si_001.pdf]

Supporting Information for
Analytical Chemistry

Quartz Crystal Microbalance as a Holistic Detector for Quantifying Complex Organic Matrices during Liquid Chromatography: 1. Coupling, Characterization, and Validation

Christopher Wabnitz[1], Aoife Canavan[1], Wei Chen[1], Mathias Reisbeck[2], and Rani Bakkour[1*]

[1]TUM School of Natural Sciences, Chair of Analytical Chemistry and Water Chemistry, Technical University of Munich, Garching 85748, Germany
[2]TUM School of Computation, Information and Technology, Heinz Nixdorf Chair of Biomedical Electronics, Technical University of Munich, Munich 81675, Germany

*Corresponding author: rani.bakkour@tum.de
phone +49 89 289 54502, fax +49 89 2180 78255

26 Pages, 5 Figures, 2 Tables

Contents

S1 Chemicals, Materials, Standard Solutions, and Spray Fabrication **2**

S2 Determination of Split Ratios **2**

S3 Microfluidic Spray Efficiency of Drying Different Solvents **3**

S4 Determination of Lower and Upper Limits of Detection and Quantification **5**

S5 TOC Validation **7**

S6 Matlab Script for QCM Data Processing **8**

S1 Chemicals, Materials, Standard Solutions, and Spray Fabrication

Table S1 List of reagents, solvents, and analytical standards.

Chemical	Purity/Grade	Supplier
sodium chloride	≥99.5 %	Fisher Scientific
acetonitrile	≥99 %	Sigma-Aldrich
methanol	≥99 %	Sigma-Aldrich

Ultrapure H_2O (18.2 MΩ cm at 25 °C) was obtained from a Milli-Q® direct reference H_2O purification system from Merck MilliPore (Burlington, USA).

The microfluidic spray-dryers were fabricated in-house at the Heinz Nixdorf-Chair of Biomedical Electronics at the Center for Translational Cancer Research of the Technical University of Munich (TranslaTUM). Fast and reliable production of microfluidic spray-dryers was achieved using a two-layer soft lithography approach according to a previously published protocol[1]. Microfluidic channels were designed in AutoCAD 2021 (Autodesk GmbH). Negative resists SU8-3025 and SU8-3050 (MicroChem Corp.) were spin-coated and patterned using a maskless laser lithography system (Dilase 250, Kloe, France) on a 3" Si substrate to obtain the negative master mold. The first layer has a thickness of 20 µm and contains the channel for the liquid sample, while the second layer has a thickness of 70 µm and contains the channel for the gas. PDMS (Sylgard 184, Dow Corning) was mixed at a ratio of 9:1 (w/w), degassed, and cured in the negative SU8 master mold for 60 min at 65 °C. Microfluidic devices were cut along alignment marks using a razor blade, and holes for tubings were punched using a 0.5 mm biopsy punch (World Precision Instruments). Individual PDMS devices were cleaned using isopropanol and acetone and blown dry with nitrogen. PDMS devices were activated using oxygen plasma for 60 s at 30 W (Zepto, Diener electronic GmbH, Germany). A drop of deionized water was applied to the surface to facilitate the alignment of two PDMS devices under a stereo microscope for the final microfluidic device. A permanent bond between PDMS parts is formed by curing the assembled devices for 60 min at 85 °C. The cross-section of the channels for liquid and gas delivery were measured to be 27 × 20 µm^2 (w × h) and 110 × 70 µm^2 (w × h), respectively.

S2 Determination of Split Ratios

Split ratios (R_{split}) were determined for different Vernier scale settings (56, 66, 73, 79, 94, and 112) for three different CH_3OH/H_2O mobile phase compositions [85/15, 50/50, and 15/85 (v/v)] by spraying the mobile phase containing 500 mg/L NaCl for 30 min into a vial. The dried salt was reconstituted in 8 mL H_2O. The salt concentration in solution was determined by measuring the salinity using a salinometer (MultiLine F/SET-3, WTW, Germany).

$$\kappa_{corrected} = \kappa_{sample} - \kappa_{control} \tag{S1}$$

$$c_{sample} = \frac{\kappa_{corrected}}{\Lambda_m^{NaCl}} \tag{S2}$$

Dividing the corrected salinity ($\kappa_{corrected}$) by the molar conductivity of NaCl (Λ_m^{NaCl}) results in the concentration of the sample in the vial (c_{sample}) (see Eq S1 and S2).

$$Q_{spray} = \frac{m_{sample}}{c_{NaCl} \cdot t} \tag{S3}$$

The flow to the spray-dryer (Q_{spray}) was calculated using Eq S3 by dividing the sprayed mass (m_{sample}), calculated using c_{sample}, by the sprayed time (t) and by the concentration of salt (C_{NaCl}) in the mobile phase.

$$R_{split} = \frac{Q_{input} - Q_{spray}}{Q_{spray}} \tag{S4}$$

The split ratio (R_{split}) was calculated using Eq S4. Here, the high flow, which is the difference of the input flow (Q_{input}) and the low flow (Q_{spray}), is divided by the low flow.

S3 Microfluidic Spray Efficiency of Drying Different Solvents

To ensure that the sprayed sample on the QCM fully dries, especially with gradient elution, a mass was deposited for 10-15 min from three different solvent compositions [H_2O/CH_3OH 9/1, 1/1, 1/9 (v/v), Figure S1]. The liquid flow is stopped (vertical dashed line), but the nitrogen stream was maintained to keep drying the QCM sensor. During only drying, no significant frequency shift was visible, which means no significant amount of residual solvent is enclosed in the deposited mass.

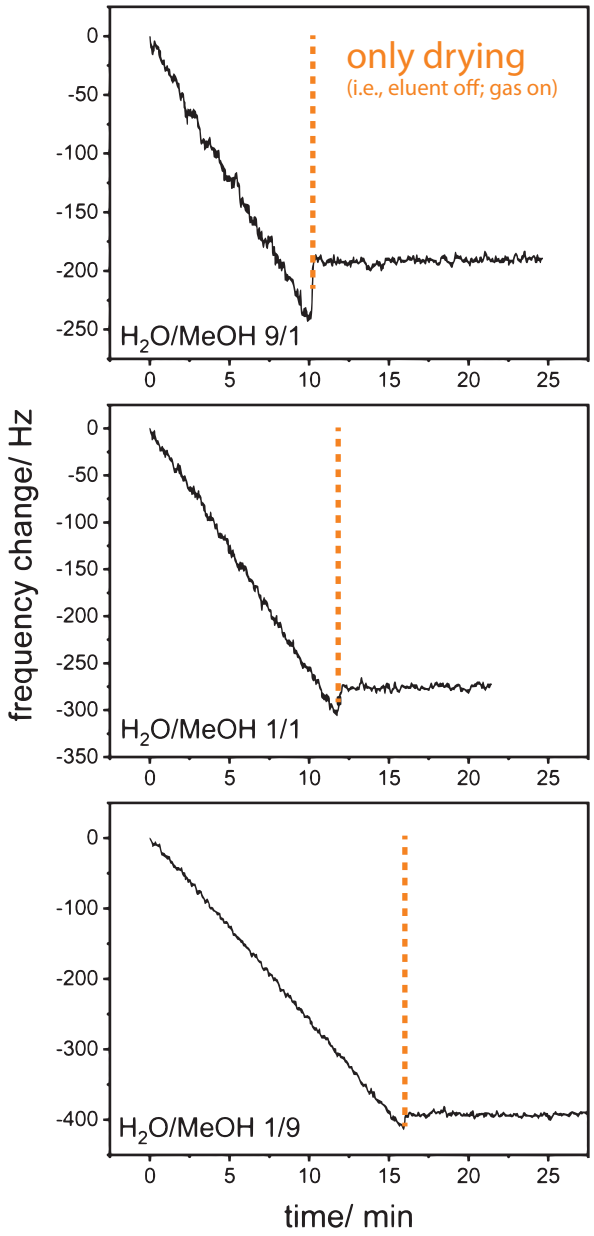

Figure S1 Dry mass sensing on QCM using microfluidic spray in different eluent compositions associated with frequency shift starting from 0 min. The orange dashed vertical line denotes a complete shut down of eluent flow whilst maintaining the flow of drying nitrogen gas.

S4 Determination of Lower and Upper Limits of Detection and Quantification

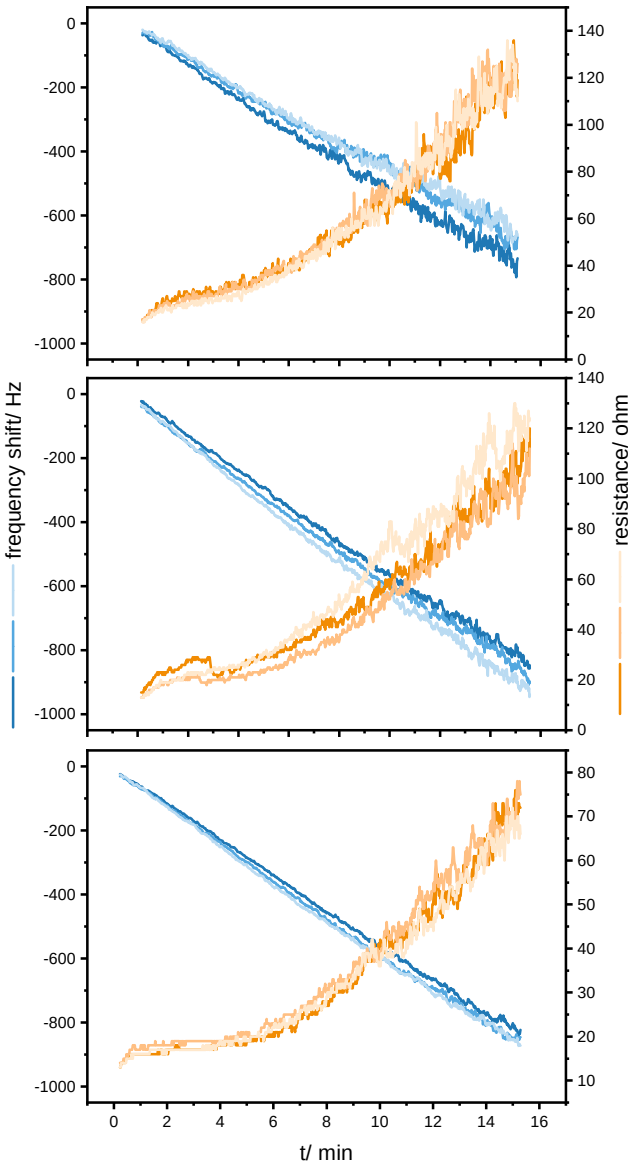

Figure S2 Frequency (blue) and resistance (orange) raw data of triplicate measurements of three different CH_3OH/H_2O compositions ((a): 15/85, (b): 50/50, (c): 85/15 (v/v)) containing 500 mg/L NaCl.

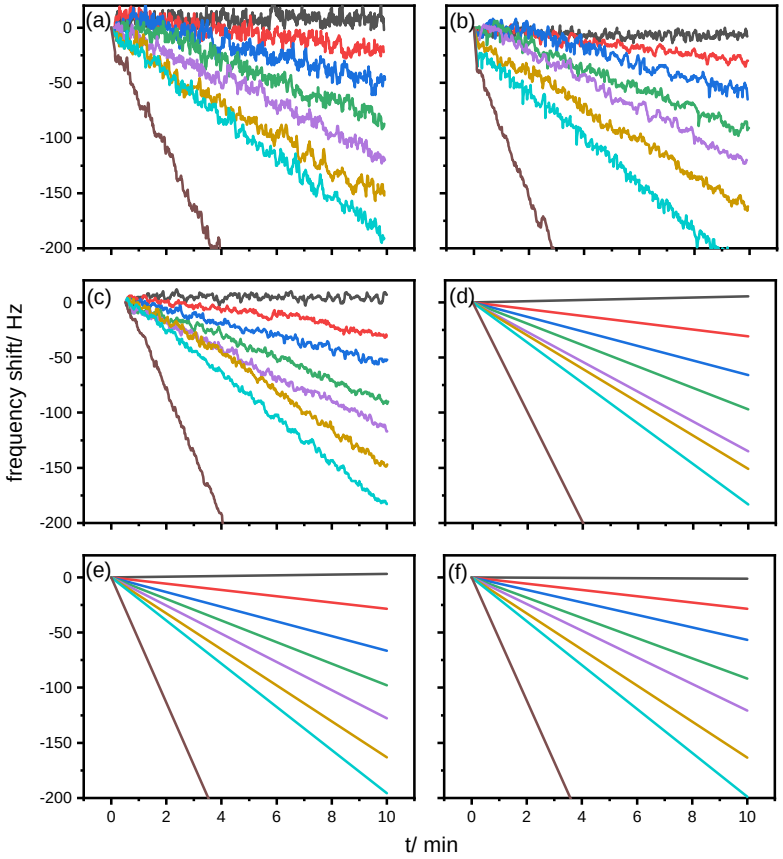

Figure S3 a-c: Frequency raw data of three different CH_3OH/H_2O compositions [(a): 15/85, (b): 50/50, (c): 85/15 (v/v)] containing NaCl in different concentrations (0, 30, 60, 90, 120, 150, 180 and 500 mg/L). (d-f): The average slope of quadruplicates of dry mass sensing experiments using different CH_3OH/ H_2O compositions [(d): 15/85, (e): 50/50, (f): 85/15 (v/v)] containing NaCl in different concentrations (0, 30, 60, 90, 120, 150, 180 and 500 mg/L). The slopes were used for the calculation of the LOD and the LOQ.

S5 TOC Validation

Table S2 HPLC gradient conditions for the TOC validation measurement.

Time/ min	% CH_3OH
0	10
7.5	40
15	80
16.5	90
18	90

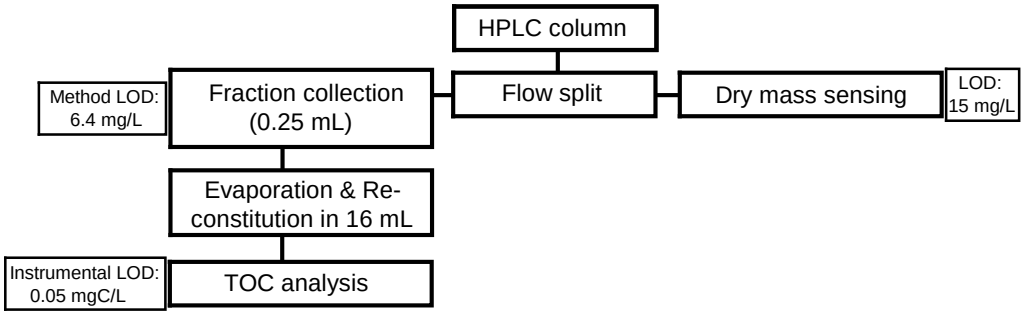

Figure S4 Flow chart of offline TOC measurement of HPLC fractions. The mobile phase is split using a post-column adjustable flow splitter. The high flow goes to a fraction collector; each fraction is collected for 30 seconds (volume: 0.25 mL). The fraction is evaporated and reconstituted in 16 mL H_2O and measured using TOC analysis.

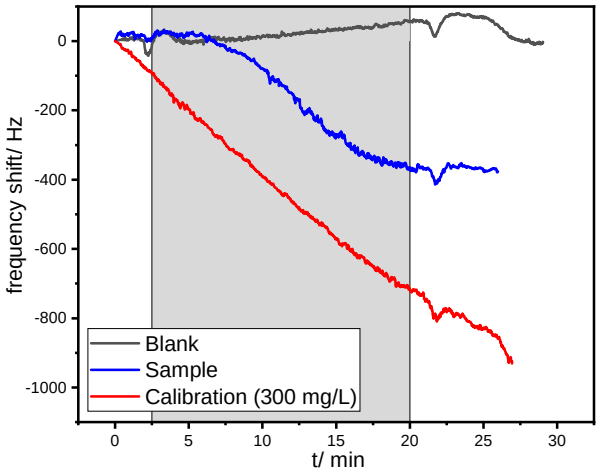

Figure S5 Frequency raw data (blank = black, sample = blue, calibration = red) of the QCM measurement during the TOC validation experiment. The grey bar shows the measurement window (2.5 min dead time).

S6 Matlab Script for QCM Data Processing

```
1
2  clc
3  clear
4
5  para.Projecttitle=('10-80-90');
6  para.Endtime=22;
7  MEOHpercent0min=10;
8  MEOHpercent75min=80;
9  MEOHpercent15min=90;
10 GradientX=[0 7.5 15 16.5 18 19 para.Endtime];
11 GradientY=[MEOHpercent0min MEOHpercent75min MEOHpercent15min 90
       90 10 10];
12
13 figure
14 plot(GradientX,GradientY,'-k')
15 title('Solvent Gradient')
16 xlabel('time /min')
17 ylabel('MeOH %')
18 xlim([0 para.Endtime])
19 ylim([0 100])
20 %%%%%%%%%%%%%%%%%%% if no QuEChERS are related QuechersBlank=0,
       otherweise
21 %%%%%%%%%%%%%%%%%%% type in any nr.
22 QuechersBlank=0;
23 UV without Blank subtraction
24 namelist=dir('*.txt');
25 len=length(namelist);
26
27 UVfilename='./UV/10_80_90.txt'%namelist(1).name;
28 % UVfilename="220324_isochratic30percent_NOMextractsample.txt";
29
30 [RetentionTime,Intensity]=textread(UVfilename,'%s%s','headerlines
       ',25);
31
32 RetentionTime=strrep(RetentionTime,',','.');
33
34 [inx_Time,~,~]=find(contains(RetentionTime,'Time'));
35 [inx_R,~,~]=find(contains(RetentionTime,'R'));
36 [inx_Wave,~,~]=find(contains(RetentionTime,'Wave'));
```

```
37
38 Wavelength=Intensity(inx_Wave);
39 Starttxt='Bandwidth(nm)';
40 Endtxt='[PDA';
41 [inx_Start,~,~]=find(contains(RetentionTime,Starttxt));
42 inx_Start=inx_Start+3;
43
44 [inx_End,~,~]=find(contains(RetentionTime,Endtxt));
45 inx_End=inx_End-1;
46 inx_End=inx_End(2:end);
47 inx_End=[inx_End;length(RetentionTime)];
48
49 for i=1:length(Wavelength)
50
51     UVtime{i}=RetentionTime(inx_Start(i): inx_End(i),1);
52     UVtime{i}= str2double(convertCharsToStrings(UVtime{i}));
53
54     UVintensity{i}=Intensity(inx_Start(i): inx_End(i),1);
55     UVintensity{i}= str2double(convertCharsToStrings(UVintensity{
          i}));
56 end
57 Raw data extraction
58
59 %%figurename must match the sequence of filelist
60 %  figurename={'Blank1', 'Blank2', 'Blank3', 'Calibration01_1','
      Calibration01_2','Calibration01_3', ...
61 % 'Calibration02_1','Calibration02_2','Calibration02_3','
      Sample01_1', 'Sample01_2', 'Sample01_3','Sample02_1', '
      Sample02_2', 'Sample02_3'};
62 para.figurename={'blank1', 'blank2', 'cali1', 'cali2', 'sample1'
      };
63
64 %%%read the file from QCM
65
66 QCMnamelist=dir('*.txt');
67 len=length(QCMnamelist);
68 n=1;
69 TableTimeDeltaF=[];
70
71 for i=1:len
```

```
72      filename{i}=QCMnamelist(i).name;
73
74      [RawF{i},   RawR{i} ,       DeltaF{i}, DeltaR{i},   DeltaMass
           {i},   Thickness{i},   Time{i},        Tag{i}]=textread(
           filename{i},'%s%s%s%s%s%s%s%s','headerlines',16);
75      DeltaF_num{i}=strrep(DeltaF{i},',','.');
76      DeltaF_num{i}=cell2mat(DeltaF_num{i});
77      %     DeltaF_num{i}=str2double(convertCharsToStrings(
           DeltaF_num{i}));
78      DeltaF_num{i}=str2num(DeltaF_num{i});
79
80      DeltaR_num{i}=strrep(DeltaR{i},',','.');
81      DeltaR_num{i}=cell2mat(DeltaR_num{i});
82      %     DeltaF_num{i}=str2double(convertCharsToStrings(
           DeltaF_num{i}));
83      DeltaR_num{i}=str2num(DeltaR_num{i});
84
85      RawR_num{i}=strrep(RawR{i},',','.');
86      RawR_num{i}=cell2mat(RawR_num{i});
87      %     DeltaF_num{i}=str2double(convertCharsToStrings(
           DeltaF_num{i}));
88      RawR_num{i}=str2num(RawR_num{i});
89
90      Time_num{i}=strrep(Time{i},',','.');
91      Time_num{i}=cell2mat(Time_num{i});
92      % Time_num{i}=str2double(convertCharsToStrings(Time_num{i}));
93      Time_num{i}=str2num(Time_num{i});
94
95      Time_min{i}=Time_num{i}./60;
96
97      % write time and delte freq to excel file.
98
99      T{i}=table(Time_min{i},DeltaF_num{i});
100
101     T{i}.Properties.VariableNames = {'Time_min','DeltaF'};
102
103     TableTimeDeltaF=T{i};
104     excelcolumn={'A2','C2','E2','G2','I2', 'K2', 'M2','O2','Q2',
           'S2', 'U2'};
105
```

```
    excelfilename = [para.Projecttitle 'TableTimeDeltaF.xlsx'];
    writetable(TableTimeDeltaF,excelfilename,'Sheet',1,'Range',
       excelcolumn{i})

    [TF{i},S1{i},S2{i}] = ischange(DeltaF_num{i},'linear','
       Threshold',30000);
end

filename
add title for excel columns

for n=1: length(para.figurename)

    excelcolumn={'A1','C1','E1','G1','I1', 'K1', 'M1','O1','Q1',
       'S1', 'U1'};

    excelfilename = [para.Projecttitle 'TableTimeDeltaF.xlsx'];
    writecell(para.figurename(n),excelfilename,'Sheet',1,'Range',
       excelcolumn{n})

end
Enter the index of blank/calibration/sample!!
blank_index=[1:2];
calibration_index=[3:4];
sample_index= [5];
Compare blanks and calibrations via figures

figure
hold on
for i=blank_index

    plot(Time_min{i},DeltaF_num{i})

end

for j=sample_index

    plot(Time_min{j},DeltaF_num{j})

end
```

```
142
143 hold off
144
145 xlim([0,25])
146 legend([para.figurename(blank_index),para.figurename(sample_index
    )],"Location",'best')
147 title('CompareBlank')
148 figure
149 hold on
150 for i=blank_index
151
152     plot(Time_min{i},RawR_num{i})
153
154 end
155
156 for j=sample_index
157
158     plot(Time_min{j},RawR_num{j})
159
160 end
161
162 hold off
163 xlim([0,para.Endtime])
164 legend([para.figurename(blank_index),para.figurename(sample_index
    )],"Location",'best')
165 title('Compare Blanks and Smaple R')
166 figure
167
168 hold on
169 for i=calibration_index
170
171     plot(Time_min{i},DeltaF_num{i})
172
173 end
174
175 for j=sample_index
176
177     plot(Time_min{j},DeltaF_num{j})
178
179 end
```

```
180
181 hold off
182
183 xlim([0,25])
184 legend([para.figurename(calibration_index),para.figurename(
       sample_index)],"Location","best" )
185 title('\DeltaF comparation')
186 ylabel('\DeltaF /Hz')
187 xlabel('time /min')
188
189 xlim([0.0 26])
190 figure
191 hold on
192 for i=calibration_index
193
194     plot(Time_min{i},RawR_num{i})
195
196 end
197
198 for j=sample_index
199
200     plot(Time_min{j},RawR_num{j})
201
202 end
203
204 hold off
205 xlim([0,para.Endtime])
206 legend([para.figurename(calibration_index),para.figurename(
       sample_index)],"Location",'best')
207 title('R-value comparation')
208 ylabel('R-value /Ohms')
209 xlabel('time /min')
210 Select Blank and Calibration
211 %%%%%Blank=3 means Blank3
212 %%%%%Cali=5 means Calibration1; C=5 or 6 or 7 for four blanks
       and three Cali
213 %------select Blank and Calibration
214 Cali=4;
215 Blank=2;
```

```
Export template file for selected calibration and sample with
   selected blank

%-------Export template file for selected calibration
Caliexcelfilename = [para.Projecttitle 'Template_Calibration.xlsx
   '];

writematrix(BLANKfinal_frequency,Caliexcelfilename,'Sheet',1,'
   Range','A1')
writematrix(Califinal_frequency,Caliexcelfilename,'Sheet',1,'
   Range','C1')

%-------Export template file for Sample

Samexcelfilename = [para.Projecttitle 'Template_Sample.xlsx'];

writematrix(BLANKfinal_frequency,Samexcelfilename,'Sheet',1,'
   Range','A1')
writematrix(SAMPLEfinal_frequency,Samexcelfilename,'Sheet',1,'
   Range','C1')
Control subtraction, filtration, derivate calculation
For Calibraition:

%-----------Load data

%Specify path:
path_to_excel_file = Caliexcelfilename;
sheet_name_in_excel = "Sheet1";
data = xlsread(path_to_excel_file, sheet_name_in_excel);

%-----------Compute the difference:
%-----------since timepoints are not aligned the difference is
   computed between two closest in time points

%define the row
time_control = 1;
frequency_control = 2;
time_calib = 3;
frequency_calib = 4;
control_timepoints = data(:,time_control);
```

```
calib_timepoints = data(:,time_calib);
control_freq = data(:,frequency_control);
calib_freq = data(:,frequency_calib);

%Specify path:
path_to_excel_file = Caliexcelfilename;
sheet_name_in_excel = "Sheet1";
data = xlsread(path_to_excel_file, sheet_name_in_excel);

%-----------Compute the difference:
%-----------since timepoints are not aligned the difference is computed between two closest in time points

%define the row
time_control = 1;
frequency_control = 2;
time_calib = 3;
frequency_calib = 4;
control_timepoints = data(:,time_control);
calib_timepoints = data(:,time_calib);
control_freq = data(:,frequency_control);
calib_freq = data(:,frequency_calib);

%%%%%%%%%%%%%%%consider the data length of Calibration
validEND=sum(~isnan(calib_freq));
if validEND>2000
    validEND=2000;
end

calib_freq=calib_freq(1:validEND);
calib_timepoints=calib_timepoints(1:validEND);
%searching for the nearest time_control and subtract control frequency from
%calibration

%searching for the nearest time_control

calib = zeros(length(calib_timepoints), 2);
for i = 1 : length(calib_timepoints)
    calib(i, 1) = calib_timepoints(i);
```

```
    [~,Index] = min(abs(control_timepoints-calib_timepoints(i)));
    calib(i, 2) = calib_freq(i) - control_freq(Index);
end

%------------------calculating derivate
%type in approx_step to adjust the timestep in regression

approx_step = 100;
derivate_data = zeros(length(calib_timepoints) - approx_step,2);
for i = 1 : (length(calib_timepoints)-approx_step)
    approx_timepoint = zeros(approx_step, 1);
    approx_frequency = zeros(approx_step, 1);
    for j = 1 : approx_step
        approx_timepoint(j) = calib(i+j-1, 1);
        approx_frequency(j) = calib(i+j-1, 2);
    end
    X = [ones(size(approx_timepoint)), approx_timepoint];
    Y = approx_frequency;
    derivate = X\Y;
    derivate_data(i, 1) = calib(i + floor(approx_step/2), 1);
    derivate_data(i, 2) = derivate(2);
end
position = "C";
position_app = position + floor((length(calib_freq)-length(
   derivate_data(:,2)))/2);
derivate_data(end,1)
%------------Applying filter
% odd num only
window_length =801;
polyorder = 3;
filt_calib = sgolayfilt(derivate_data, polyorder, window_length);
filt_calib(end,1)
figure
plot(calib(:, 1), calib(:, 2), '-r', derivate_data(1:end,1),
   derivate_data(1:end,2), '-b')
hold on
plot(filt_calib(1:end,1), filt_calib(1:end,2),'-k','LineWidth',2)
hold off
xlim([0 27])
```

```
legend('\DeltaF', '1st Derivate', 'Filtered Derivate','Location'
   ,"best")
title('Calibration', 'Data processing')
xlabel('time /min')
ylabel({'Frequency /Hz'; '1^st Derivate of \DeltaF'})
plot( derivate_data(:,1), derivate_data(:,2), '-b')
hold on
plot(filt_calib(:,1), filt_calib(:,2),'-k','LineWidth',2)
hold off
ylim([-200 150])
xlim([0 27])
x0=10;
y0=10;
width=1200;
height=400;
set(gcf,'position',[x0,y0,width,height])
For Sample:
path_to_excel_file = Samexcelfilename;
sheet_name_in_excel = "Sheet1";
data = xlsread(path_to_excel_file, sheet_name_in_excel);

%-----------Compute the difference:
%-----------since timepoints are not aligned the difference is
   computed between two closest in time points

%define the row
time_control = 1;
frequency_control = 2;
time_calib = 3;
frequency_calib = 4;
control_timepoints = data(:,time_control);
calib_timepoints = data(:,time_calib);
control_freq = data(:,frequency_control);
calib_freq = data(:,frequency_calib);

%searching for the nearest time_control

calib = zeros(length(calib_timepoints), 2);
for i = 1 : length(calib_timepoints)
    calib(i, 1) = calib_timepoints(i);
```

```
    [~,Index] = min(abs(control_timepoints-calib_timepoints(i)));
    calib(i, 2) = calib_freq(i) - control_freq(Index);
end

%------------------calculating derivate
%type in approx_step to adjust the timestep in regression

approx_step = 30;
derivate_data = zeros(length(calib_timepoints) - approx_step,2);
for i = 1 : (length(calib_timepoints)-approx_step)
    approx_timepoint = zeros(approx_step, 1);
    approx_frequency = zeros(approx_step, 1);
    for j = 1 : approx_step
        approx_timepoint(j) = calib(i+j-1, 1);
        approx_frequency(j) = calib(i+j-1, 2);
    end
    X = [ones(size(approx_timepoint)), approx_timepoint];
    Y = approx_frequency;
    derivate = X\Y;
    derivate_data(i, 1) = calib(i + floor(approx_step/2), 1);
    derivate_data(i, 2) = derivate(2);
end
position = "C";
position_app = position + floor((length(calib_freq)-length(
   derivate_data(:,2)))/2);

%------------Applying filter
window_length = 301;
polyorder = 3;
filt_SAMPLE = sgolayfilt(derivate_data, polyorder, window_length)
   ;

%------------plot data
figure
plot(calib(:, 1), calib(:, 2), '-r', derivate_data(:,1),
   derivate_data(:,2), '-b')
hold on
plot(filt_SAMPLE(:,1), filt_SAMPLE(:,2),'-k','LineWidth',2)
hold off
xlim([0 27])
```

```
399 legend('\DeltaF', '1st Derivate', 'Filtered Derivate','Location'
       ,"best")
400 title('Sample', 'Data processing')
401 xlabel('time /min')
402 ylabel({'Frequency /Hz'; '1^st Derivate of \DeltaF'})
403 %------------zoom
404
405 plot( derivate_data(:,1), derivate_data(:,2), '-b')
406 hold on
407 plot(filt_SAMPLE(:,1), filt_SAMPLE(:,2),'-k','LineWidth',2)
408 hold off
409 ylim([-200 150])
410 xlim([0 20])
411 x0=10;
412 y0=10;
413 width=1200;
414 height=400;
415 set(gcf,'position',[x0,y0,width,height])
416 %-----------------saving data to the file
417 %first row is time
418 %second row is calb.frequency
419
420 head = {'time_calib', 'frequency_calib'};
421 file_name = "Sample_Frequency-filtered.xlsx";
422 if exist(file_name, 'file') == 1
423     delete(file_name);
424 end
425 writematrix(calib, file_name, 'range', 'A2');
426 writematrix("time_calib",file_name, 'range', 'A1');
427 writematrix("frequency_calib",file_name, 'range', 'B1');
428 writematrix("filtered gravity", file_name, 'range', 'C1');
429 writematrix(derivate_data(:, 2), file_name, 'range', position_app
       )
430 Correction of time (shift, cut off)
431 df_dm=0.1415;
432
433 %-------input timeshift
434 timeshift=-2.50;
435
436
```

```
shifted_filt_calibtime=filt_calib(:,1)+timeshift;

shifted_filt_SAMPLEtime=filt_SAMPLE(:,1)+timeshift;

%////////cut off the negative timeframe

cutted_shifted_filt_calibtime=shifted_filt_calibtime(
   shifted_filt_calibtime>0 & shifted_filt_calibtime<20);

cutted_shifted_filt_SAMPLEtime=shifted_filt_SAMPLEtime(
   shifted_filt_SAMPLEtime>0 & shifted_filt_SAMPLEtime<20);

cutted_shifted_filt_calibfreq=filt_calib(:,2);
cutted_shifted_filt_calibfreq=cutted_shifted_filt_calibfreq(
   shifted_filt_calibtime>0 & shifted_filt_calibtime<20);

cutted_shifted_filt_SAMPLEfreq=filt_SAMPLE(:,2);
cutted_shifted_filt_SAMPLEfreq=cutted_shifted_filt_SAMPLEfreq(
   shifted_filt_SAMPLEtime>0 & shifted_filt_SAMPLEtime<20);

figure
plot(cutted_shifted_filt_SAMPLEtime,
   cutted_shifted_filt_SAMPLEfreq)
Gradient construction: run if HPLC with Gradient
GRADIENT.startMEOH=0.111;

%%%%%%%%%%%%%%%%% y=mx+b
GRADIENT.m1=5.2952;
GRADIENT.m2=3.7;
GRADIENT.m3=11.1;

GRADIENT.t1=8.4524;
GRADIENT.t2=26;
GRADIENT.t3=-77.6;

%-------input sample concentrations in H2O and MeOH

GRADIENT.sample_meoh=300;
GRADIENT.sample_h2o=300;

```

```
gradientcomposition=zeros(length(cutted_shifted_filt_calibtime),1);
gradientcomposition(cutted_shifted_filt_calibtime<=0.5)= GRADIENT.startMEOH;
gradientcomposition(cutted_shifted_filt_calibtime>0.5 & cutted_shifted_filt_calibtime<=11)=(cutted_shifted_filt_calibtime(cutted_shifted_filt_calibtime>0.5 & cutted_shifted_filt_calibtime<=11).* GRADIENT.m1 + GRADIENT.t1)/100;
gradientcomposition(cutted_shifted_filt_calibtime>11 & cutted_shifted_filt_calibtime<=14)=(cutted_shifted_filt_calibtime(cutted_shifted_filt_calibtime>11 & cutted_shifted_filt_calibtime<=14).* GRADIENT.m2 + GRADIENT.t2)/100;
gradientcomposition(cutted_shifted_filt_calibtime>14 & cutted_shifted_filt_calibtime<=16) = (cutted_shifted_filt_calibtime(cutted_shifted_filt_calibtime>14 & cutted_shifted_filt_calibtime<=16).* GRADIENT.m3 + GRADIENT.t3)./100;
gradientcomposition(cutted_shifted_filt_calibtime>16) = 1;

plot(cutted_shifted_filt_calibtime,gradientcomposition)
figure
yyaxis left
plot(cutted_shifted_filt_calibtime,gradientcomposition.*90)
ylim([0 100])

yyaxis right
plot(CaliDF(:,1), CaliDF(:,2));

legend('Solvent Gradient','Calibration \DeltaF','Location',"south")
xlim([0 20])
concentration_sprayed=ones(length(cutted_shifted_filt_calibtime),1);

concentration_sprayed=gradientcomposition.*GRADIENT.sample_meoh + (1-gradientcomposition).* GRADIENT.sample_h2o;

figure
```

```
495 plot(cutted_shifted_filt_calibtime,concentration_sprayed)
496 Calculation of sprayed concentration
497
498 Isocratic: Run if isocratic, input the solvent composition of
       MeOH
499
500 %  Isocratic!!+++++++++calculation of sprayed conc.
501 %%% deactive  if with Gradient
502 % GRADIENT.sample_meoh=290.6055516;
503 % GRADIENT.sample_h2o=290.2358244;
504 %
505 % gradient_HPLC=0.3333;
506 %
507 % concentration_sprayed=GRADIENT.sample_meoh.*gradient_HPLC+
       GRADIENT.sample_h2o.*(1-gradient_HPLC);
508 sample concentration calculated
509 if length(cutted_shifted_filt_calibfreq)< length(
       cutted_shifted_filt_SAMPLEfreq)
510     cutted_shifted_filt_SAMPLEfreq=cutted_shifted_filt_SAMPLEfreq
          (1:length(cutted_shifted_filt_calibfreq));
511     cutted_shifted_filt_SAMPLEtime=cutted_shifted_filt_SAMPLEtime
          (1:length(cutted_shifted_filt_calibfreq));
512     concentration_sprayed=concentration_sprayed(1:length(
          cutted_shifted_filt_calibfreq));
513 else
514     cutted_shifted_filt_calibfreq=cutted_shifted_filt_calibfreq
          (1:length(cutted_shifted_filt_SAMPLEfreq));
515     cutted_shifted_filt_calibfreq=cutted_shifted_filt_calibfreq
          (1:length(cutted_shifted_filt_SAMPLEtime));
516     concentration_sprayed=concentration_sprayed(1:length(
          cutted_shifted_filt_SAMPLEfreq));
517 end
518
519 %--------------------sample concentration calculated
520
521 concentration_sample_passedColumn=cutted_shifted_filt_SAMPLEfreq
       ./cutted_shifted_filt_calibfreq.*concentration_sprayed;
522
523 %/////////plot
524
```

```
%%%%%%Mass Calculation

para.MassInteEndtime=20;

validtimeframe=cutted_shifted_filt_SAMPLEtime>=0 &
    cutted_shifted_filt_SAMPLEtime<=para.MassInteEndtime &
    concentration_sample_passedColumn>=0;

SprayTotalMass=trapz(cutted_shifted_filt_SAMPLEtime(
    validtimeframe),concentration_sample_passedColumn(
    validtimeframe))/2000
SprayTotalMass = 1.9785
TheoInjectedNOM=1.80;
MassBalance=SprayTotalMass/TheoInjectedNOM
FinalResult=table(cutted_shifted_filt_SAMPLEtime,
    concentration_sample_passedColumn);

% % % % % % % % % % % % % % % % % % % % % export excel

FinalResult=table(cutted_shifted_filt_SAMPLEtime,
    concentration_sample_passedColumn);

FinalResult.Properties.VariableNames = {'Time_min','Mass Conc. /
    mg/L'};

TableFinalResult=FinalResult;
excelcolumn={'A2','C2','E2','G2','I2', 'K2', 'M2','O2','Q2', 'S2'
    , 'U2','W2', 'Y2'};

excelfilename = [para.Projecttitle 'TableFinalResult.xlsx'];
writetable(TableFinalResult,excelfilename,'Sheet',1,'Range',
    excelcolumn{1})

f = figure;
u = f.Units;
f.Units = 'normalized';

% % legend([p1 p3],{'First','Third'})

xlim([0,20])
```

```
556
557 g_1=gca;
558
559 plot_1=plot(cutted_shifted_filt_SAMPLEtime,
      concentration_sample_passedColumn, 'LineWidth',2,"Color",'k');
560 ylim ([-10,700])
561 xlim([0,20])
562 g_1.YColor='k';
563 xlabel('Time /min');
564 % legend('QCM')
565
566 Y1=ylabel('Mass /mg');   label
567 xlim_1=get(g_1,'xlim');
568 ylim_1=get(g_1,'ylim');
569
570 pos_2=get(g_1,'position');
571 g_2=axes('Position',pos_2,'Color','none','XTick',[],'
      YAxisLocation','right');
572 Y2=ylabel('UV-Vis');   label
573 g_2.YColor='k';
574 hold on;
575 plot_2=plot(UVtime{1}, UVintensity{1},'-','LineWidth',1,"Color",'
      #0072BD');
576
577 xlim([0,20])
578 % ylim ([0,100])
579 pos_1=g_1.Position;
580 pos_1(1)=pos_1(1)-0.03;
581 pos_1(3)=pos_1(3)*0.9;
582
583 g_3=axes('Position',pos_1,'Color','none','XTick',[],'YTick',[]);
584 set([g_1;g_2;g_3],'position',pos_1);
585 hold on;
586
587 plot_3=plot(GradientX,GradientY,'-','LineWidth',1,"Color", '#
      D95319');
588 % legend('Gradient: MeOH %')
589
590 xlim([0,20])
591 ylim ([0,100])
```

```
pos_4=pos_1;
pos_4(3)=pos_4(3)+0.1;
g_4=axes('Position',pos_4,'Color','none','XTick',[],'YLim',[0
    100],'YTick',[0:10:100],'YAxisLocation','right');

Y3=ylabel('MeOH %');  label
legend([plot_1,plot_2, plot_3], {'QCM','220nm PestMix','Gradient:
    MeOH %'}, 'Location', "northwest","Box","off")

g_4.YColor='k';
grid off;
title(para.Projecttitle)
%%%%%%%%%%%%%%%%%%%%PestMix peak time
Peaktime=[];
Peaktime(1,:)=[6.258 6.883];
Peaktime(2,:)=[6.700 7.317];
Peaktime(3,:)=[6.983 7.575];
Peaktime(4,:)=[8.125 8.675];
Peaktime(5,:)=[9.183 9.750];
Peaktime(6,:)=[9.908 10.433];
Peaktime(7,:)=[10.125 10.783];
Peaktime(8,:)=[10.367 10.908];

for i=1:length(Peaktime)
    Peak_Starttime=Peaktime(i,1);
    Peak_Endtime=Peaktime(i,2);
    Peaktimedata=cutted_shifted_filt_SAMPLEtime>=Peak_Starttime&
        cutted_shifted_filt_SAMPLEtime<=Peak_Endtime;
    Spray_Peak_NOMMass(i)=trapz(cutted_shifted_filt_SAMPLEtime(
        Peaktimedata),concentration_sample_passedColumn(
        Peaktimedata))/2000;

end
Spray_Peak_NOMMass
RelNOM_Peak=Spray_Peak_NOMMass/SprayTotalMass*100
```

References

[1] Kartanas, T.; Ostanin, V. P.; Challa, P. K.; Daly, R.; Charmet, J.; Knowles, T. P. J. Enhanced Quality Factor Label-free Biosensing with Micro-Cantilevers Integrated into Microfluidic Systems. *Anal. Chem.* **2017**, *89 22*, 11929–11936.
